# Leech removal is not the primary driver of basking behavior in a freshwater turtle

**DOI:** 10.1002/ece3.7876

**Published:** 2021-08-01

**Authors:** Donald T. McKnight, Wytamma Wirth, Lin Schwarzkopf, Eric J. Nordberg

**Affiliations:** ^1^ College of Science and Engineering James Cook University Townsville Qld Australia; ^2^ College of Public Health, Medical and Veterinary Sciences James Cook University Townsville Qld Australia; ^3^ School of Environmental and Rural Science University of New England Armidale NSW Australia

**Keywords:** aerial basking, ectoparasite, parasite, reptile, thermoregulation

## Abstract

Leaving the water to bask (usually in the sun) is a common behavior for many freshwater turtles, with some species also engaging in “nocturnal basking.” Ectoparasite removal is an obvious hypothesis to explain nocturnal basking and has also been proposed as a key driver of diurnal basking. However, the efficacy of basking, day or night, to remove leeches has not been experimentally tested. Therefore, we examined the number of leeches that were removed from Krefft's river turtles (*Emydura macquarii krefftii*) after experimentally making turtles bask at a range of times of day, durations, and temperatures. Turtles had high initial leech loads, with a mean of 32.1 leeches per turtle. Diurnal basking under a heat lamp for 3 hr at ~28°C significantly reduced numbers of leeches relative to controls. In diurnal trials, 90.9% of turtles lost leeches (mean loss of 7.1 leeches per turtle), whereas basking for 30 min under the same conditions was not effective (no turtles lost leeches, and all turtles were still visibly wet). Similarly, “nocturnal basking” at ~23°C for 3 hr was not effective at removing leeches. Only 18% of turtles lost leeches (one turtle lost one leech and another lost four leeches). Diurnal basking outdoors under direct sunlight for 20 min (mean temp = 34.5°C) resulted in a small reduction in leeches, with 50% of turtles losing leeches and an average loss of 0.7 leeches per turtle. These results indicate basking can remove leeches if temperatures are high or basking durations are long. However, it was only effective at unusually long basking durations in this system. Our data showed even the 20‐min period was longer than 70.1% of natural diurnal basking events, many of which took place at cooler temperatures. Therefore, leech removal does not appear to be the purpose of the majority of basking events.

## Graphical abstract

Aerial basking has long been hypothesized to reduce ectoparasites in turtles. We experimentally tested if basking reduced leech loads in an Australian freshwater turtle. While basking can reduce leech loads, the basking duration and heat intensity greatly influence the results. Thus, we have determined that mean basking times in nature were not sufficient to effectively reduce leech loads, making leech removal unlikely to be the primary mechanism driving basking behavior.

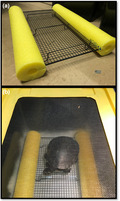

## 1 INTRODUCTION

Ectoparasites can adversely impact host health by absorbing nutrients and spreading diseases (Bower et al., 2019). As a result, parasites often influence host ecology and behavior, including causing hosts to shift behaviors in an effort to remove parasites or mitigate the diseases they spread (Schall & Sarni, 1987; Main & Bull, 2000, Bower et al., 2019). Birds, for example, engage in behaviors such as preening, dust bathing, and sunning to remove parasites (Bush & Clayton, 2018).

Leeches are a common ectoparasite for many freshwater turtles, and they can transmit hemoparasites, such as the apicomplexan protozoa of the genera *Haemogregarina* and *Hepatozoon* (Strohlein & Christensen, 1984; Siddall & Desser, 1992; Rossow et al., 2013; Arizza et al., 2016). This may have important consequences, because there is some evidence that high parasitemia of red blood cells can result in anemia or other histopathologies (Schall et al., 1982; Peirce & Adlard, 2007; Despommier et al., 2017). Therefore, it may be advantageous for turtles to engage in behaviors that remove parasites; however, the fitness costs have not been well established (see Schall, 1986 and Brown et al., 2006).

Basking behavior in turtles is generally thought to be thermoregulatory (specifically, increasing body temperature) and is often referred to as “sunning” due to turtles’ proclivity for sitting on sunny substrates with their limbs outstretched (Boyer, 1965; Chessman, 1987, 2020). Some authors have, however, challenged the notion that basking is primarily for thermoregulation (e.g., Manning & Grigg, 1997). To further complicate matters, some turtles “bask” at night (Barhadiya et al., 2020; Nordberg & McKnight, 2020), a time period when this behavior would not result in increased body temperatures from direct solar radiation. Nocturnal basking may, however, provide an opportunity to thermoregulate by avoiding unfavorable water temperatures (Nordberg and McKnight pers. obs.).

An alternative hypothesis proposes that basking is a mechanism for removing ectoparasites, particularly leeches (Mcauliffe, 1977; Koffler et al., 1978; Reshk, 2009; Mitchell & Johnston, 2012). Given that the leeches that parasitize turtles are aquatic, this is a reasonable proposal, and there are scattered, anecdotal observations of leeches leaving basking turtles (Saumure & Livingstone, 1994; Selman et al., 2008; Selman & Qualls, 2009). Conversely, Vogt (1979) anecdotally reported finding live leeches attached to turtles that had been kept out of water for four days, and Hall (1922) reported that the leech *Placobdella parasitica* can survive desiccation up to a body water loss of ~92%. The effect basking has on leeches will likely depend on factors such as the body size of leeches (larger leeches should desiccate more slowly), temperature, basking duration, and the leech attachment locations on the host.

Most studies that have attempted to test the parasite removal hypothesis have done so by comparing parasite loads among species that do and do not bask regularly (Mcauliffe, 1977; Strohlein & Christensen, 1984; Siddall & Desser, 1992; Ryan & Lambert, 2005; Gaertner et al., 2008; Readel et al., 2008; Davis & Sterrett, 2011; Rossow et al., 2013). These studies had mixed results, high levels of variation, and, often, low sample sizes, but they generally reported higher parasite loads in species that bask less frequently. However, as many of the authors of those studies argued, the species being compared differed greatly in many ways beyond basking activity, and other factors such as leech preference, time spent on the bottom of waterways, and amount of exposed body area likely offer better explanations for the observed differences in parasite loads (Siddall & Desser, 1992; Ryan & Lambert, 2005; McCoy et al., 2007; Gaertner et al., 2008).

Arguably, the most compelling evidence that basking behavior relates to parasites comes from Ibanez et al. (2015). They found that individuals infected with *Hepatozoon* spp. spent more time basking than did uninfected individuals, which they interpreted as a form of behavioral fever. While this is a rational interpretation, Mitchell and Johnston (2012) found that individuals captured while basking had fewer leeches than individuals captured in the water, and they suggested that reduced parasite loads in basking individuals could indicate that basking removes parasites. Thus, these studies do not present a falsifiable approach, and both possible results (i.e., basking turtles have more parasites or basking turtles have fewer parasites) have been interpreted as supporting the hypotheses that basking removes parasites. This mix of results from observational studies makes it clear that experimental studies are needed.

In this study, we experimentally tested whether freshwater turtles can use aerial basking to effectively reduce leech loads. Individuals in our study population frequently bask nocturnally (Nordberg & McKnight, 2020) as well as diurnally; therefore, we conducted both diurnal and nocturnal trials. Additionally, we provided much‐needed data on leech prevalence and intensity in an Australian turtle.

## 2 METHODS

### 2.1 Study site and species

We conducted this study in Ross River, Townsville, Queensland, in northeastern Australia. The Ross River is predominantly lined with *Melaleuca* spp. and *Eucalyptus* spp. trees, as well as shrubs, logs, and aquatic vegetation. The river supports a large population of Krefft's river turtles (*Emydura macquarii krefftii*) which are frequently parasitized by the leech *Placobdelloides bancrofti* (McKenna et al., 2005) and can easily be observed basking during both the day and night (Nordberg & McKnight, 2020).

### 2.2 Natural basking durations

**FIGURE 1:**
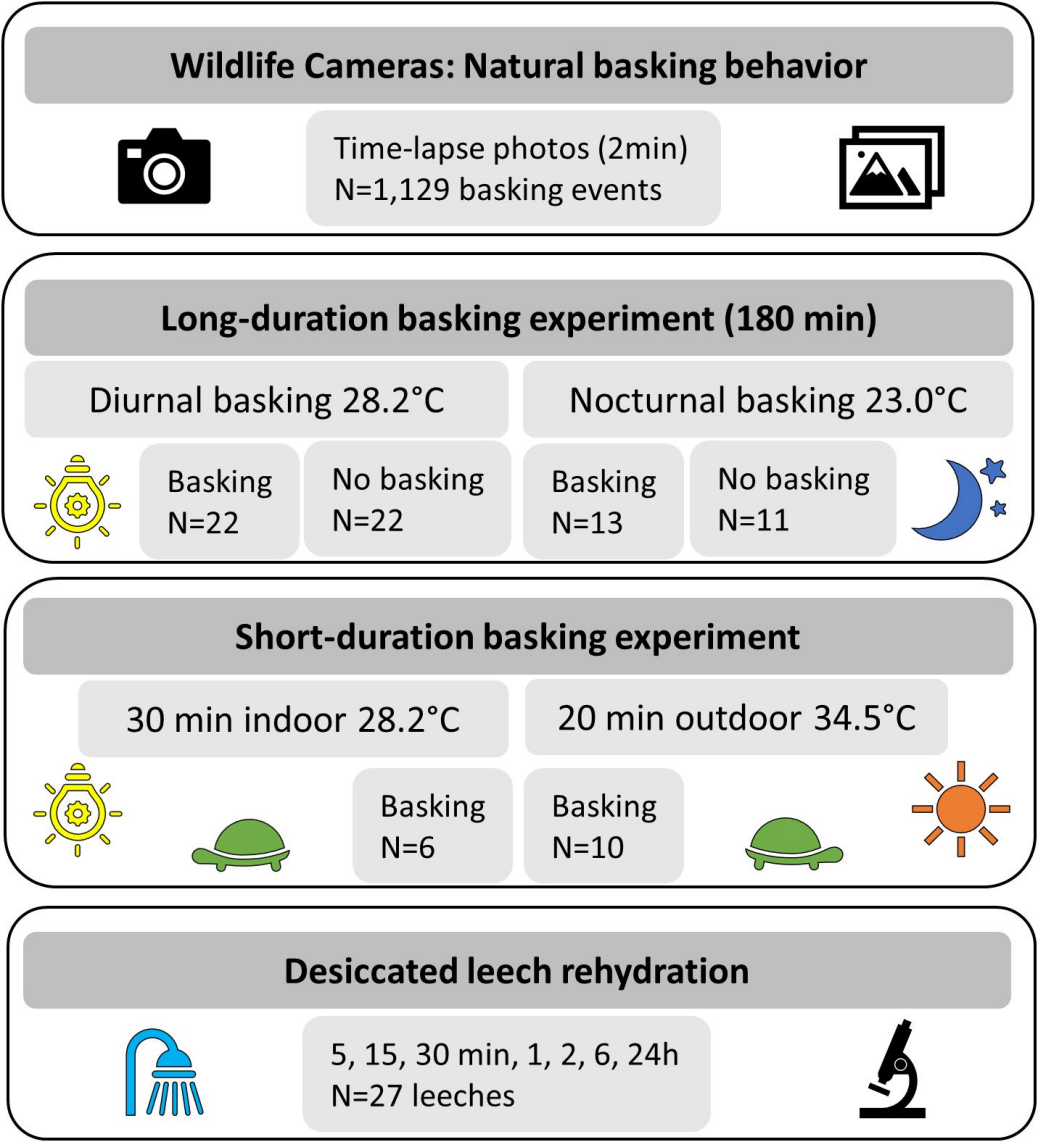
Outline of the experiments used in this study

To estimate normal turtle basking durations, we deployed three wildlife trail cameras (Campark T85) on separate basking logs in the Ross River from 11–17 Nov 2020 (Figure 1, Figure S1). Mean air temperature during this period was 30.1°C during the day and 25.2°C during the night. The cameras took a photograph every two minutes, including taking photographs at night using an IR flash. We used these photographs to record the duration of each basking event and separated basking events into “night” and “day” based on sunrise and sunset times. Each time a turtle left the water, it was scored as a new event; some individuals may have provided more than one event.

### 2.3 Turtle capture and leech counts

We used sardine‐baited cathedral traps to capture turtles in and at the mouth of a small creek feeding into the Ross River. We captured turtles over three time periods: 17 Nov to 21 Dec 2020 for the long‐duration diurnal experiment, 11–25 Feb 2021 for the nocturnal experiment, and 23 Mar 2021 for short‐duration diurnal trials (individuals for the short‐duration tirals were captured slightly further up the creek than most individuals in previous samples). We transported turtles to James Cook University (5‐min drive) individually in damp cloth bags to keep leeches from desiccating. To ensure all turtles had wet skin and all leeches were fully hydrated, we placed all turtles in tubs of dechlorinated water for 1 hr prior to starting the experiments.

Immediately prior to starting a trial for a given turtle, we counted the leeches on the shell (carapace and plastron), anterior body (head, neck, front legs, and leg sockets), and posterior body (tail, neck, rear legs, and leg sockets; Brooks et al., 1990). Immediately after each trial, we counted the total number of dead leeches on the turtle, as well as the number of leeches that had detached and were found in the water. Live leeches generally appeared moist and were moving or would respond to touch with tweezers, whereas dead leeches were tightly curled into hard, dried balls and were unresponsive. To ensure that these leeches were truly dead and not just severely desiccated, we placed them in water for 5–45 min to give them a chance to rehydrate. We only scored them as “dead” if they did not reanimate. We returned all turtles to their capture locations within 24 hr of initial capture.

To look for associations between the initial leech load and the sex and size of the turtles, we used a negative binomial model. We used the initial leech load (i.e., the total number of leeches at the start of the trial) as the response variable and the capture period (Nov–Dec, Feb, or Mar), sex, and curved carapace length (CCL) as the predictor variables. Because females are much larger than males, we wanted to first look for an overall difference in the sexes, then look at size after accounting for sex. Therefore, we built the model with the factors in the order listed and used an ANOVA with a type I sum of squares. This tested for differences in the sexes after accounting for differences in capture periods and tested for associations with CCL after accounting for both capture periods and sexes. We constructed and tested the model in R using base functions (v4.0.3; R Core Team, 2017).

To look for differences in leech loads among body regions, we used a mixed effects negative binomial model in the R package lme4 (v1.1‐26; Bates et al., 2015). We included initial leech load as the response variable, capture period, body region, and sex (with an interaction with body region) as the fixed effects, and turtle ID as a random intercept. We used the Anova function in the car package (v3.0–10; Fox & Weisberg, 2011) with a type II sum of squares to test significance (thus testing each factor given the other factors). We used the emmeans package to perform post hoc Tukey tests (v1.5.4; Lenth, 2018). Due to a significant interaction, we compared regions separately within males and within females, and we compared males and females separately for each region.

### 2.4 Long‐duration basking experiment (diurnal and nocturnal)

**FIGURE 2:**
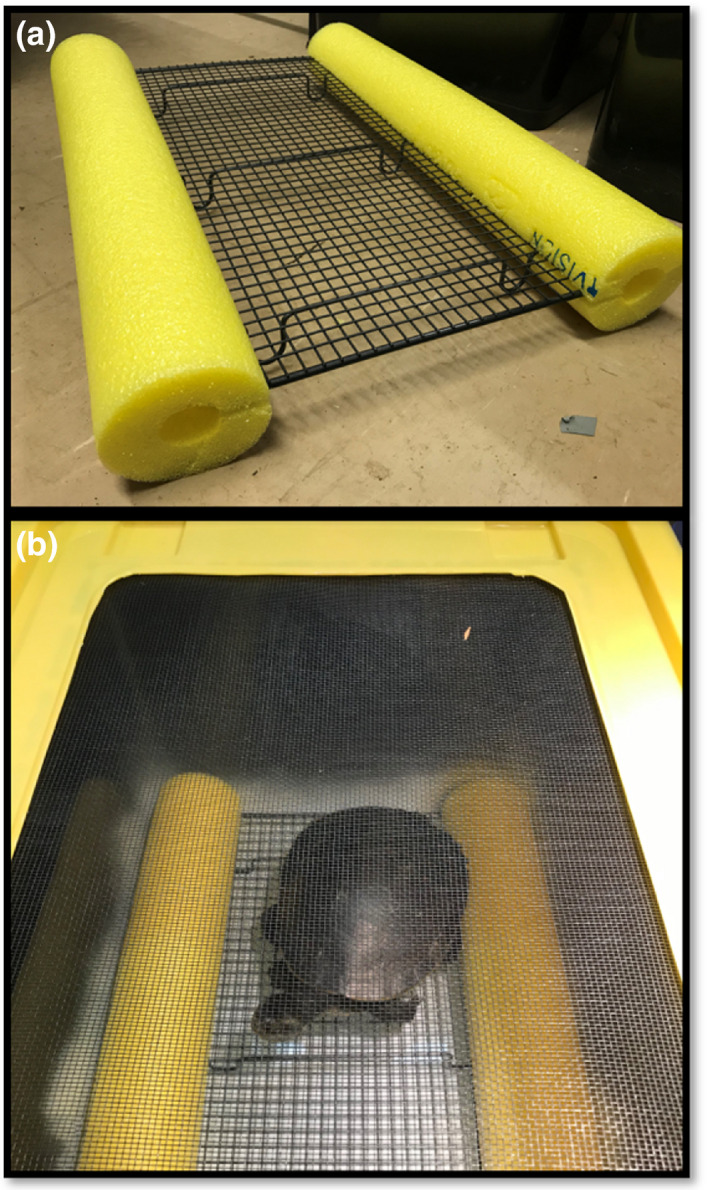
Basking platform made from a baking rack and pool noodle (a) cut to size and placed inside the testing arena to deny turtle access to the water (b). Nonbasking turtles were placed in a similar testing arena filled with water but without the basking platform

We tested whether aerial basking was an effective behavioral mechanism to reduce leech loads in freshwater turtles. Our experiment included two basking treatments: 1: nonbasking—turtles remained in tubs filled with water with no access to a basking platform (this ensured that turtles would remain in the water) and 2: basking—turtles were elevated out of the water on a small mesh baking rack to simulate a turtle basking on a structure over water (Figure 2). Water was present below the basking platform to prevent leeches from desiccating if they detached from a turtle and fell into the water or migrated down the legs of the tray.

Because *E. m. krefftii* in this population bask at night, we conducted both diurnal and nocturnal trials. Diurnal trials started between 09:00 and 12:00, and nocturnal trials started between 18:00 and 19:20. For the diurnal trials, we suspended an 80‐watt heat lamp above a pair of testing arenas (one basking and one nonbasking arena per pair) to simulate the thermal conditions experienced by turtles basking in the sun. No heat lamps were used for nocturnal trials, and turtles were kept in the dark to simulate natural conditions. All trials were conducted in a temperature and light‐cycle controlled room. Average ambient temperature in the room was 22.7°C (*SD* = 0.37°C), and basking temperatures under the heat lamp were 28.2°C (*SD* = 1.04°C). Water temperatures were not recorded in each trial, but during the first trial, the water temperature had equilibrated to the air temperature (~22°C) which would have also occurred for all subsequent trials. Ambient room temperatures during nocturnal trials were similar (mean = 23.0°C, *SD* = 0.87°C). The room humidity was 50.9% on average (*SD* = 11.3%). Each trial lasted for 180 min. We deliberately chose this time to represent the extreme end of basking durations to maximize our chances of detecting an effect. We tested up to 12 turtles simultaneously (each in a separate testing arena) depending on how many were captured on a given day (diurnal and nocturnal trials took place on separate days with separate batches of turtles). On each day, we randomized turtles into one of the two treatments.

To statistically analyze the results, we compared the four treatments using a negative binomial model with the number of leeches that were dead or in the water at the end of the trial as the response variable, treatment as the predictor variable, and initial leech load as a covariate. We assessed significance with a type II ANOVA via the Anova function in the car package.

### 2.5 Short‐duration basking experiments (diurnal)

Following the long‐duration experiment, we conducted two additional experiments to test the effects of shorter basking periods and the intensity of basking temperatures. The first of these experiments was identical to the long‐duration diurnal basking treatment, but it ran for only 30 min. We terminated these trials after only six individuals (see results).

The second experiment was designed to look at the effects of a shorter basking interval with a greater heat intensity. Again, testing arenas were the same as the long‐duration diurnal basking treatment, but turtles were placed outside in full sunlight (all on 23 Mar 2021). We used masking tape to cover and temporarily attach Thermochron iButton temperature data loggers (Maxim Integrated Products) to each turtle's carapace to monitor the external temperature (recorded every 2 min during the trials; mean = 34.5°C, *SD* = 2.9°C, range = 29.0–40.5°C; recorded temperatures increased over the course of the trials as turtles’ shells warmed). We monitored turtles closely for signs of heat stress and only conducted trials for 20 min to avoid overheating the turtles. All potentially dead leeches from this trial were kept in water for a full 24 hr to ensure they were truly dead, and they were only included as dead in the results if they did not recover within 24 hr.

Based on the results of the long‐duration diurnal basking experiment, we did not include controls for either short‐duration experiment and only determined the ability of short‐duration basking events to remove leeches. To statistically analyze the results of the high‐intensity heat experiment, we made comparisons to the diurnal nonbasking and diurnal basking results from the long‐duration experiment (each tested with a separate model). In other words, we compared short‐duration, high‐intensity basking with long‐duration, low‐intensity basking, and we compared short‐duration, high‐intensity basking with the long‐duration control. The latter test was admittedly crude because the control turtles were checked after three hours and the basking turtles were checked after 20 min; however, even after three hours, only two leeches (both from the same turtle) had fled the control turtles and none had died, so it is unlikely that a shorter period would have produced substantially different results. For each test, we used a negative binomial model with the number of dead or removed leeches as the response, treatment as the predictor, and initial leech load as a covariate. We assessed significance with type II ANOVAs via the Anova function in the car package.

### 2.6 Leech dehydration

Following the long‐duration experiment, we ran a small trial to ensure that our criteria for determining that leeches were dead were sufficient. For this trial, we captured seven additional *E. m. krefftii* and exposed them to a 180‐min diurnal basking period (identical to the long‐duration diurnal basking trial) for the sole purpose of collecting and testing “dead” leeches (we did not measure these turtles or perform initial leech counts and they were not included in any summary statistics). We collected 27 leeches that appeared dead from these turtles, placed them in vials of water, and checked them for signs of life at 5, 15, 30 min, 1, 2, 6, and 24 hr (“reanimated” leeches moved around and adhered to the walls of the vial, whereas dead leeches remained motionless at the bottom of the vial).

## 3 RESULTS

### 3.1 Natural basking durations

**FIGURE 3:**
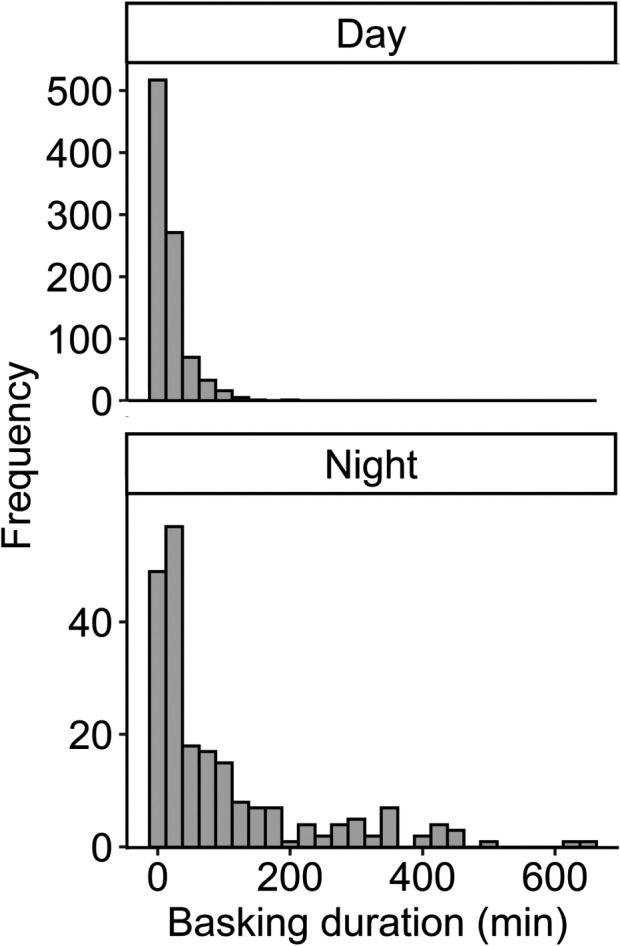
Basking frequencies for *Emydura macquarii krefftii* over a 7‐day monitoring period in November 2020. Basking frequency and duration were monitored via remote wildlife cameras, capturing photographs on time‐lapse mode, taking photographs every 2 min for 7 days. Histogram bins = 25 min

We documented 914 diurnal basking events, lasting for a mean of 19.0 min (*SD* = 22.4, median = 10, range = 2–195) and 215 nocturnal basking events, lasting for a mean of 100.2 min, (*SD* = 130.1, median = 39, range = 2–652; Figure 3; Figure S2). An additional 55 basking events transitioned between night and day (duration [min]: mean = 205.1, *SD* = 195.6, median = 146, range = 4–774) and were not included in the above calculations due to difficulty classifying them as diurnal or nocturnal basking events. However, most of the transitional basking events that included long basking events started in the late afternoon or early evening (diurnal) and remained out of the water throughout the night (nocturnal). Our long‐duration basking experiment (180 min) was longer than 99.9% of diurnal basking events and 82.8% of nocturnal basking events. Our 30‐min indoor experiment and 20‐min outdoor experiment were longer than 81.8% and 70.1% of observed diurnal basking events, respectively.

### 3.2 Leech loads and turtle data

**FIGURE 4:**
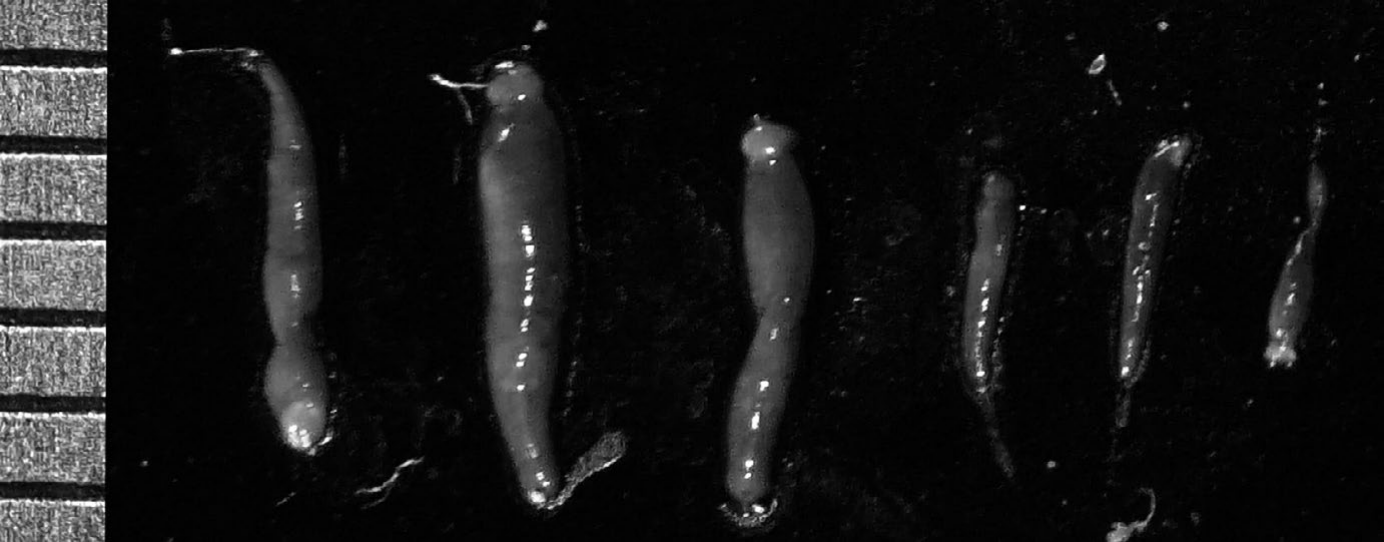
Six representative leeches illustrating the normal sizes of leeches on our turtles. Each tick mark = 1 mm

**FIGURE 5:**
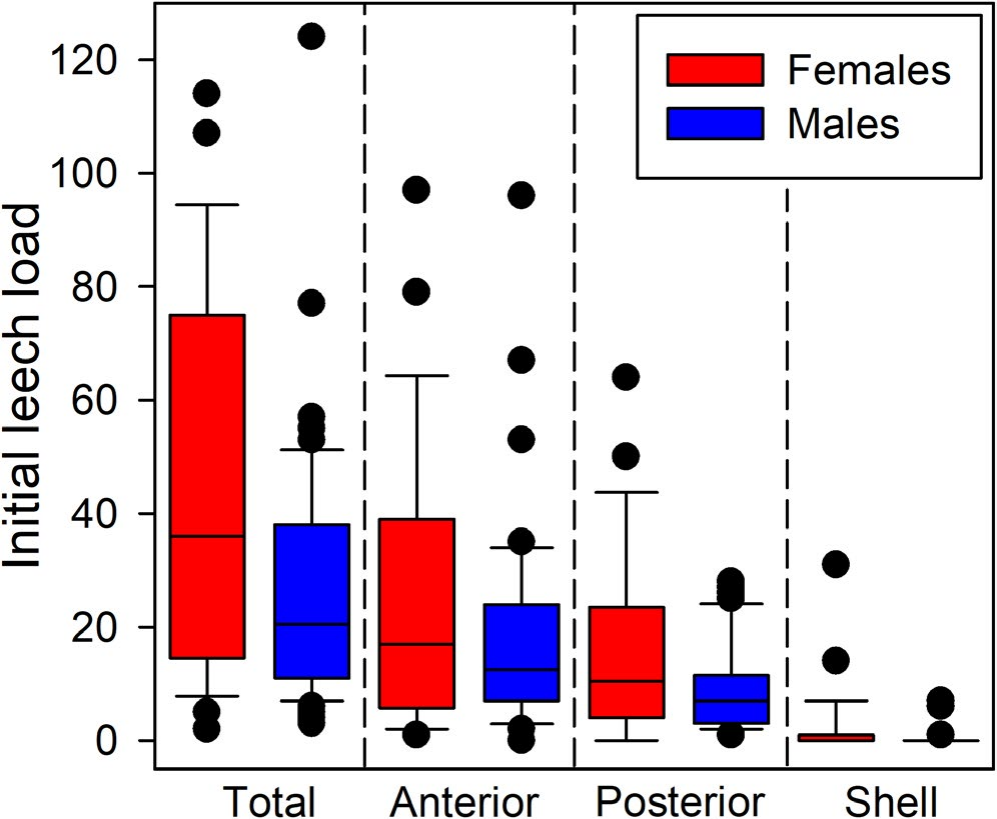
Number of leeches at the start of the experiment, separated by body region and sex. Whiskers represent the 10th/90th percentile, and all outliers are shown

Leeches (*Placobdelloides bancrofti*) ranged in size from ~1–8 mm (Figure 4). Leeches were unevenly distributed on turtles. Very few were found on the shell, and these were almost entirely restricted to the carapace (Figure 5). They frequently formed clumps of individuals, often hiding in skin folds, and they generally stayed proximal to the body, rather than being spread evenly across the extremities.

Turtles had a high leech prevalence and often high loads. In total, we captured 85 turtles, only one of which did not have any leeches and was excluded from all tests and summary statistics (it was a male from the first capture period, CCL = 225 mm, mass = 1,030 g). The 84 turtles with leeches had an average of 32.1 leeches (*SD* = 26.3, range = 2–124). Females had significantly more leeches than males (*F*_(1,80)_ = 4.904, *p* = 0.027), but there was no significant relationship between initial leech load and turtle size (*F*_(1,78)_ = 0.035, *p* = 0.852). There was a significant difference among capture periods (*F*_(1,81)_ = 14.829, *p* < 0.001); turtles captured during the final period had the highest leech loads. It is unclear if this was a seasonal effect or a result of the slight difference in capture locations. Full morphometric data and leech counts are available in Table 1.

**TABLE 1:** Mean (*SD*) turtle morphometrics and initial leech counts. Data are presented for each capture period and for all capture periods combined: 1 = 17 Nov to 21 Dec 2020, used for long‐duration diurnal trials, 2 = 11–25 Feb 2021, used for nocturnal trials, 3 = 23 Mar 2021, used for short‐duration diurnal trials (collected slightly further up a creek than most other samples)

Sex	Trapping period 1			Trapping period 2			Trapping period 3			Total		
	F	M	Total	F	M	Total	F	M	Total	F	M	Total
*N*	13	31	44	5	19	24	8	8	16	26	58	84
CCL	234.3 (27.1)	206.9 (13.1)	215 (22)	247.4 (37.4)	211.8 (17.2)	219.2 (26.3)	255.3 (15.6)	218 (13.3)	237.9 (23.8)	243.3 (27.1)	209.9 (14.9)	220.3 (4.3)
Mass	1,374.6 (470.2)	875.3 (174.7)	1,022.8 (368.9)	1654 (655.6)	940.7 (228.9)	1,089.3 (450.9)	1717.5 (312.6)	922.1 (144.4)	1,346.3 (475.9)	1533.8 (478.7)	902.9 (190.7)	1,100.5 (425.9)
# of leeches	38.7 (31.8)	21.3 (14.1)	26.4 (22)	18 (16.8)	30.3 (27.4)	27.8 (25.8)	68.8 (27)	37.7 (21.2)	54.3 (28.5)	44.0 (32.9)	26.3 (20.8)	32.1 (26.3)

CCL, curved carapace length (mm). Mass is reported in grams.

The mixed effects model confirmed that there was a significant difference among the leech loads on different body regions (*χ* ^2^ = 240.067, *p* < 0.001) and a difference between sexes (χ^2^ = 7.594, *p* = 0.006) as well as an interaction between body region and sex (*χ* ^2^ = 10.904, *p* = 0.004). Post hoc Tukey tests (which accounted for multiple comparisons in the P value calculations; separated by sex or region) showed that all regions differed within males (all *p* < 0.001), and for females, the shell had significantly fewer leeches than the anterior (*p* < 0.001) or posterior (*p* < 0.001) region of skin, but the posterior and anterior regions were not significantly different (*p* = 0.219). When comparing sexes within each body region, females consistently had higher mean leech loads, but the difference was only significant for the shell (*p* < 0.001). It was nearly significant for the posterior region (*p* = 0.054) and nonsignificant for the anterior region (*p* = 0.316).

### 3.3 Long‐duration basking experiment (diurnal and nocturnal)

**FIGURE 6:**
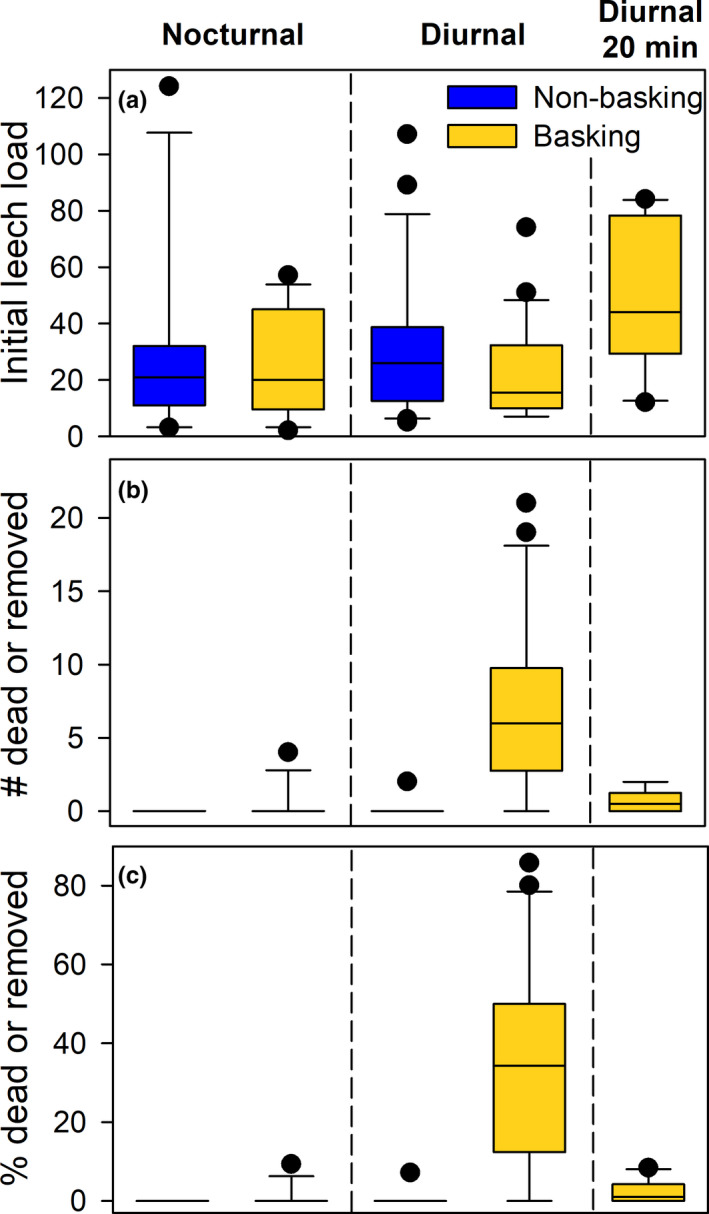
Effects of treatments on leeches. (a) Number of leeches at the start of the experiment, (b) number of leeches that were removed (dead or in the water) by the end of the experiment, (c) percent of initial leech load that was removed by the end of the trial. Results are shown for the long‐duration nocturnal and diurnal basking experiments, and the 20‐min outdoor diurnal basking experiment. Whiskers represent the 10th/90th percentile, and all outliers are shown

There was a significant difference in the number of leeches that were removed by the basking treatments (χ^2^_(3,63)_ = 154.391, *p* < 0.001) and a significant positive relationship between the number of leeches that were removed and the initial leech load (*χ* ^2^_(1,63)_ = 8.948, *p* = 0.003). The difference among treatments was driven by the diurnal basking treatment (Figure 6). Of 22 turtles in the diurnal basking treatment, 20 (90.9%) lost at least one leech, with an average loss of 7.1 leeches per turtle (*SD* = 6.1; 95% CI = 4.6–9.6), whereas only one turtle (of 22) in the diurnal nonbasking (control) group lost leeches (it lost two leeches). This suggests that diurnal basking for long durations can remove leeches. In contrast, only two turtles (of 13) in the nocturnal basking group lost leeches (one and four), and no turtles (of 11) in the nocturnal nonbasking (control) group lost leeches, suggesting that long‐duration nocturnal basking is minimally effective at removing leeches. Most leeches desiccated and died (*n* = 164) rather than fleeing (*n* = 11), but on three occasions (all during diurnal basking trials) live leeches were found in the water at the end of the trial (*n* = 2, 3, 6).

### 3.4 Short‐duration basking experiments (diurnal)

During the indoor 30‐min basking trials, all turtles still had visibly wet skin at the end of the trial, and there was no evidence of leech desiccation on any turtle. Therefore, we terminated the experiment after six trials, because it was clear that such a short duration at that heat intensity was insufficient to dry turtles and cause leeches to desiccate.

At the end of our high heat intensity outdoor experiment (20 min), five turtles had lost zero leeches, three turtles had one dead leech each, and two turtles had two dead leeches each, a mean loss of 0.7 leeches (*SD* = 0.82) across all ten turtles. This was a significantly smaller reduction in leech load than in the long‐duration (180 min) basking experiment (χ^2^_(1,29)_ = 36.593, *p* < 0.001), but significantly more than the control from the long‐duration (180 min) basking experiment (χ^2^_(1,29)_ = 6.485, *p* = 0.011). One individual in this trail also had a midge fly larva on the skin near the tail. This larva was still alive and on the turtle at the end of the trial.

### 3.5 Leech rehydration experiment

In our leech rehydration experiment, only 6 of 27 leeches recovered. Their recoveries were first visible at the following times: one at five minutes, two at 15 min, one at one hour, one at six hours, one at 24 hr. Therefore, the majority of leeches that we categorized as dead in our primary basking experiment were truly dead, and few recoveries would have taken place outside of the rehydration period we used in the experiment. Any recordings of “death” that were false should have been minimal (~7%–11% of recorded deaths) and would not impact our conclusions.

## 4 DISCUSSION

### 4.1 Basking and leech removal

To our knowledge, this study provides the first experimental test of the hypothesis that basking removes leeches from turtles. Our results show that basking can remove leeches if temperatures are high or basking durations are long; however, the amount of time required for effective parasite removal was substantially longer than most basking events by turtles in this population. Indeed, our 20‐min outdoor experiment under natural conditions for a Townsville summer only resulted in the removal of 0.7 leeches per turtle (on average), and only half the turtles lost leeches, even though our trial was longer than 70.1% of the natural diurnal basking events we observed in November. Further, a previous assessment of basking behavior in this population found a mean basking duration of 17.4 min in winter and 8.4 min in summer (mean air temp during basking = 25.3°C; Drane, 2019). Thus, even our shortest trial was relatively long (20 min) and hot (mean = 34.5°C), and, based on these results, most natural basking events would have little or no effect on the number of leeches on a turtle.

Our data do suggest that turtles bask for longer intervals at night than during the day, but the duration of our nocturnal experiment was still fairly extreme, with an interval longer than 82.8% of observed nocturnal basking events. Therefore, while basking can remove leeches under some conditions, most basking events (both diurnal and nocturnal) are not long enough to be effective for leech removal, and a need for leech removal likely does not explain the majority of basking events for this population.

This result makes sense, given that leeches can endure high levels of desiccation. Indeed, the leech *Placobdella parasitica* can survive a loss of body water content up to ~92% (Hall, 1922). Therefore, turtles would have to bask for extended periods to desiccate leeches to a lethal level. In our indoor trials at 28.2°C, turtles’ skin was still visibly wet after basking for 30 min, and even in the outdoor experiment with direct sunlight and a mean temperature of 34.5°C, turtles’ skin was still visibly wet after 10 min, partially wet at 15 min, and just starting to dry completely at 20 min. Even at 20 min, there were still pockets of moisture in skin folds, and leeches tended to clump together (often in those folds), thus limiting desiccation by reducing the surface area exposed to the air.

Nevertheless, mean turtle basking durations can vary based on factors such as species, climate, and time of year, and there are many reports of mean basking durations longer than what we observed. Two caveats should be discussed. First, there is often a negative relationship between temperature and basking duration or frequency (Hammond et al., 1988; Lefevre & Brooks, 1995; Selman & Qualls, 2011; Drane, 2019), and reduced temperatures also result in longer desiccation times. Thus, our observed basking periods were short due to our hot, tropical climate, but regions with longer basking periods will also tend have longer desiccation times due to cooler climates. Second, even for species in cooler climates, mean basking times are often well under the 180 min we used in our long‐duration trials (the only ones to remove leeches with a reasonable level of efficacy). For yellow‐blotched sawbacks (*Graptemys flavimaculata*) in Mississippi (USA), mean basking durations in the winter were nearly 60 min, and in the summer, they were usually <30 min and often <20 min (Selman & Qualls, 2011). Similarly, most basking events for painted turtles (*Chrysemys picta*) near the northern extend of their range (Ontario, Canada) are <60–100 min long (Schwarzkopf & Brooks, 1985; Lefevre & Brooks, 1995; Carrière et al. 2008). It would be useful to repeat our experiment on additional species in different climates to determine whether mean basking times ever correspond with the time required to desiccate leeches on turtles.

There are anecdotal reports of leeches fleeing basking turtles (e.g., Selman et al. 2008; Selman & Qualls, 2009), but that was not the typical behavior we observed (though it did occur in three trials). Leeches displayed negative phototaxis to even the low‐intensity LED light we used for counting, and on several occasions during trials, leeches left the carapace and moved into a leg socket, but in general, they tended to form clumps in moist folds in a turtle's skin (often where the skin met the shell) rather than actually abandoning the turtle. Given that leeches can survive high levels of desiccation and most basking events are short (in relation to desiccation periods), it is probably more advantageous for leeches to simply seek refuge in a moist crevice and wait for the turtle to return to the water, rather than fleeing and being forced to find a new host.

Although we performed tests over several time periods, temperatures, and photoperiods (i.e., diurnal or nocturnal), there are clearly many factors that can affect drying time (e.g., humidity, temperature, and turtle posture), and there are several additional caveats that should be discussed. First, nearly all our leeches were found in the leg sockets, where they received the greatest protection against desiccation. Few leeches were present on the shell or distal ends of the limbs. Although leeches are generally less common on the carapace than on soft tissues (Dodd, 1988; Brooks et al., 1990), other studies have reported higher proportions of leeches on the carapace than we observed (Readel et al., 2008), and there are reports of leeches being more abundant on the shell than body (Reshk, 2009). Leeches on the carapace would, presumably, be at a higher risk of desiccation due to direct sun exposure. Conversely, turtles with high algal accumulation on the carapace may further shelter leeches from desiccation.

Second, turtles in our study often sat with their limbs on the substrate (i.e., not tucked into the shell) and frequently moved around the arenas, but did not assume a characteristic basking posture, with legs and neck fully outstretched. That posture would maximize skin exposure and, by stretching the skin, reduce the number of locations where leeches could seek refuge from desiccation. Thus, it could reduce the amount of time required to kill leeches, but based on our observations, we do not think it would substantially alter the results, and individuals in this population frequently bask without assuming that posture (Nordberg and McKnight pers. obs.).

Leech size is another factor to consider. Our leeches were very small (Figure 4), and larger leeches will take longer to desiccate. Indeed, adult *Placobdella parasitica*, which are common on North American turtles, are substantially larger than the leeches we observed. Brooks et al. (1990) reported that *Placobdella parasitica* on common snapping turtles (*Chelydra serpentina*) ranged from 2–80 mm. In contrast, adult *Placobdelloides bancrofti* have a maximum length of ~8 mm and width of 2 mm (McKenna et al., 2005), and most of the leeches on our turtles were very small. Thus, turtles with large leeches may require substantially longer basking intervals than the ones we tested to remove leeches.

Finally, our sample sizes for this study were admittedly small, and this is a caveat that certainly should be considered; however, the results were clear, with low enough variation to provide a reasonable level of confidence. Indeed, for our long‐duration diurnal trial, our result that basking removed a mean of 7.1 leeches per turtle had a 95% confidence interval of 4.6–9.6. Similarly, in the long‐duration nocturnal trial, only two of our 13 turtles lost any leeches (one leech, and four leeches). That is, such a consistent result that it is unlikely that a larger sample size would shift it substantially enough to alter our conclusions. The same is true for our other trials (see Figure S3).

Our conclusion that most basking events have minimal impacts on leeches is counter to the common view that basking is effective at removing leeches (Mcauliffe, 1977; Koffler et al., 1978; Reshk, 2009, Mitchell & Johnston, 2012). However, the data supporting that hypothesis come almost entirely from anecdotal observations and comparisons among species with different levels of basking behavior. In contrast, our study tested the hypothesis by experimentally comparing the influence of basking behavior on leeches within a turtle species. Further, as many previous authors have noted, species used in the cross‐species comparisons differ in many other important ways such as amount of exposed skin and the ability to molt scutes (Siddall & Desser, 1992; Ryan & Lambert, 2005; McCoy et al., 2007; Gaertner et al., 2008); thus, species comparisons are entirely confounded by other aspects of the species’ ecology and morphology. Additionally, for some of the species used in those comparisons, leech preference experiments have shown that leeches preferentially choose the “nonbasking” species (Araya, 2016; Ryan & Lambert, 2005). The reason for this preference is unclear, and it is possible that, while basking is not particularly effective at removing leeches, it is still disadvantageous for leeches, resulting in a selection pressure for leeches to choose species that bask less frequently.

It is additionally worth noting that the hypothesis that turtles bask to remove leeches hinges on the premise that there is a fitness cost to infection by leeches (or the hemoparasites they carry). The evidence supporting that premise is, however, limited and mixed. In western fence lizards (*Sceloporus occidentalis*), infection with a malarial parasite (*Plasmodium mexicanum*) results in significant reductions in hemoglobin concentrations, aerobic scope, and running stamina (consequences that would potentially be serious for breath‐holding turtles; Schall et al., 1982). Similarly, water pythons (*Liasis fuscus*) that are heavily parasitized with *Hepatozoon* sp. exhibit reduced body condition and growth (Madsen et al., 2005). However, a study on keelback snakes (*Tropidonophis mairii*) did not find any associations between hemogregarine parasites and a suite of host fitness measures (e.g., body condition, growth, and locomotor performance; Brown et al., 2006), and a study of Aruban whiptail lizards (*Cnemidophorus arubensis*) found that only resting oxygen consumption (but not active oxygen consumption, sprint distance, or endurance distance) differed between lizards that were and were not infected with hemogregarine parasites (Schall, 1986). Nevertheless, many species of animal clearly have evolved anti‐parasite behaviors (Bush & Clayton, 2018), and in an evolutionary arms race between parasites and hosts, we might expect a stable equilibrium of behaviors that allow low or moderate infections while preventing extreme infections that have serious host fitness costs. It would be fruitful for future studies to look more closely at the fitness costs of parasites in turtles.

### 4.2 Leech infection intensity

In addition to testing the effectiveness of basking for removing leeches, we also provided data on leech infection intensity and prevalence in *E. m. krefftii*. Out of our 85 turtles, 98.8% were infected, and 92.9% had more than five leeches. Few studies have examined leech prevalence and intensity in Australian turtles (Chessman, 1987; Saumure & Doody, 2000; Tucker et al., 2005), but most have found much lower infection intensities. A study of northern red‐faced turtles (*Emydura australis*) and sandstone snake‐necked turtles (*Chelodina burrungandjii*) in the Kimberly (AU) found leech prevalence of 0%–5.5% and 12.3%–83.3%, respectively (results varied by site and year; Tucker et al., 2005). Similarly, a study of *E. macquarii* in Victoria (AU) reported a prevalence of 34.5%, and only 10.7% of turtles had an infection intensity greater than five leeches (Chessman, 1987).

Females in our study had more leeches than did males. Several other studies have reported a higher prevalence or intensity of leeches (or the hemogregarine parasites they carry) in females than in males (McCoy et al. 2007; Davis & Sterrett, 2011), but not all studies agree (Dodd, 1988; MacCulloch, 1981). A common explanation for this occurrence is that females are often larger than males, and several studies have found positive relationships between body size and leech intensity or prevalence (Dodd, 1988; Readel et al., 2008). Body size differences may also explain our results, because females were larger than males on average; however, there was no significant relationship between size and leech intensity after accounting for the differences between the sexes. Our result that leeches are more common on the skin than on the shell is consistent with most other studies (Dodd, 1988; Brooks et al. 1990; Readel et al., 2008; but see Reshk, 2009); however, those studies generally found higher leech loads on the posterior portion of the turtles (inguinal area and caudal tissue), whereas we found a higher abundance on the anterior region around the neck and front legs. The reason for this difference is unclear.

## 5 CONCLUSION

Our study provides the first experimental evidence that basking could serve as a method to remove ectoparasites living on freshwater turtles; however, the amount of time required to effectively remove leeches *via* desiccation was substantially longer than most basking intervals used by turtles in our population during either the day or night. Therefore, it does not appear that leech removal is the primary reason for diurnal or nocturnal basking behavior in this population (i.e., it does not explain average basking events). Nevertheless, it is possible that some turtles, particularly turtles with high leech loads, could choose to bask for abnormally long intervals for the purpose of parasite removal. Indeed, basking may be a complex, context‐dependent behavior with multiple interacting costs and benefits. This study addresses a long‐standing hypothesis regarding an important aspect of turtle ecology and behavior and represents an important advance in our knowledge, but more research is needed to continue studying the drivers of basking behavior, including investigating the causes, costs, and benefits of nocturnal basking, as well as examining the ability of basking to remove leeches in other turtle species and climates.

## CONFLICT OF INTEREST

The authors have no conflicts of interest to declare.

## AUTHOR CONTRIBUTIONS

**Donald T. McKnight:** Conceptualization (equal); Data curation (equal); Formal analysis (lead); Investigation (equal); Project administration (equal); Supervision (equal); Writing‐original draft (equal); Writing‐review & editing (equal). **Wytamma Wirth:** Data curation (equal); Investigation (equal); Writing‐original draft (equal); Writing‐review & editing (equal). **Lin Schwarzkopf:** Project administration (equal); Resources (equal); Supervision (equal); Writing‐review & editing (equal). **Eric J. Nordberg:** Conceptualization (equal); Data curation (equal); Funding acquisition (lead); Investigation (equal); Methodology (equal); Project administration (equal); Resources (equal); Supervision (equal); Visualization (equal); Writing‐original draft (equal); Writing‐review & editing (equal).

## Supporting information

Figure S1‐S3

## Data Availability

Data are accessible in the Dryad repository (McKnight et al., 2021).
