# Supplementary material for: Leech removal is not the primary driver of basking behavior in a freshwater turtle
Source: Ecol Evol. 2021 Aug 1;11(16):10936–46. doi: 10.1002/ece3.7876 (PMC8366836; doi:10.1002/ece3.7876)
Supplement: Supplementary file 1 — Figure S1‐S3 [file ECE3-11-10936-s001.docx]

Supplemental information for Donald McKnight, Wytamma Wirth, Lin Schwarzkopf, Eric Nordberg. Leech removal is not the primary driver of basking behavior in a freshwater turtle


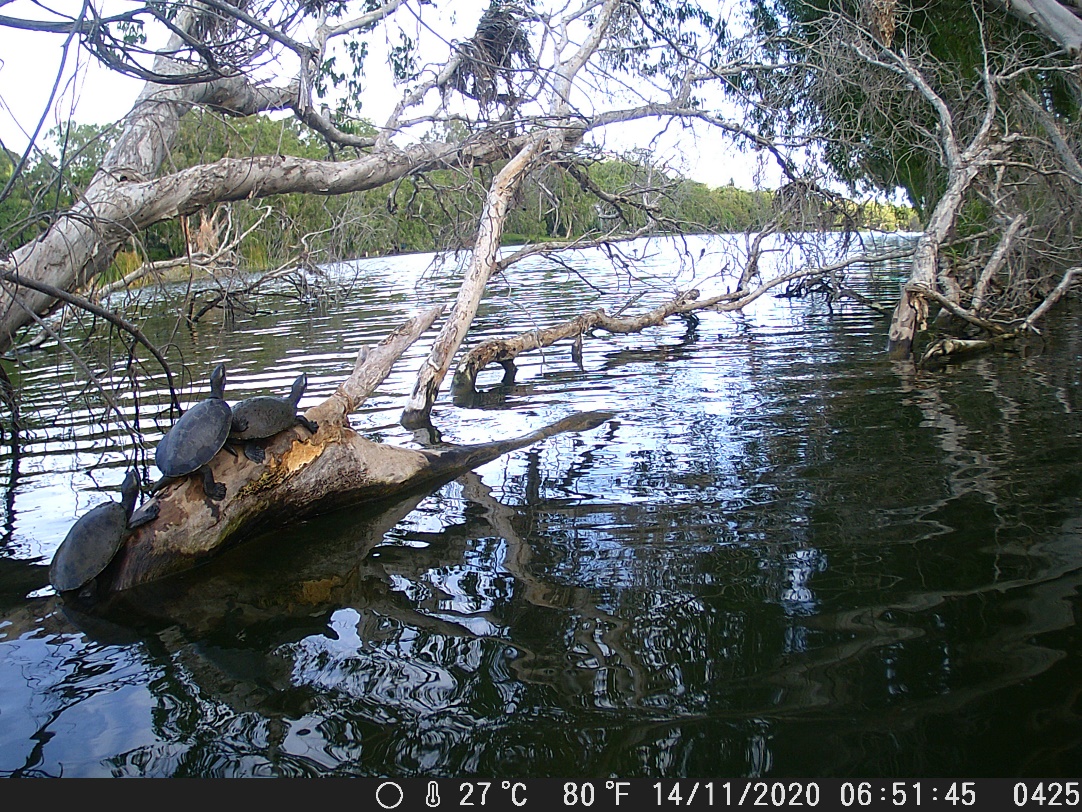


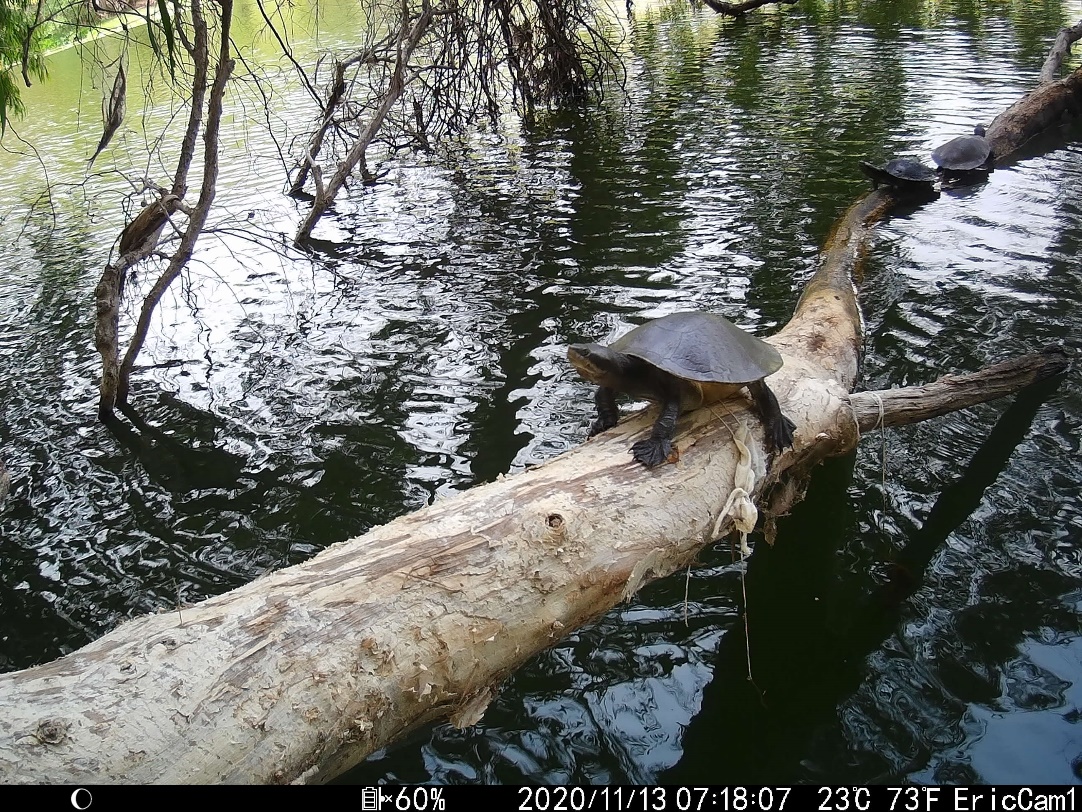


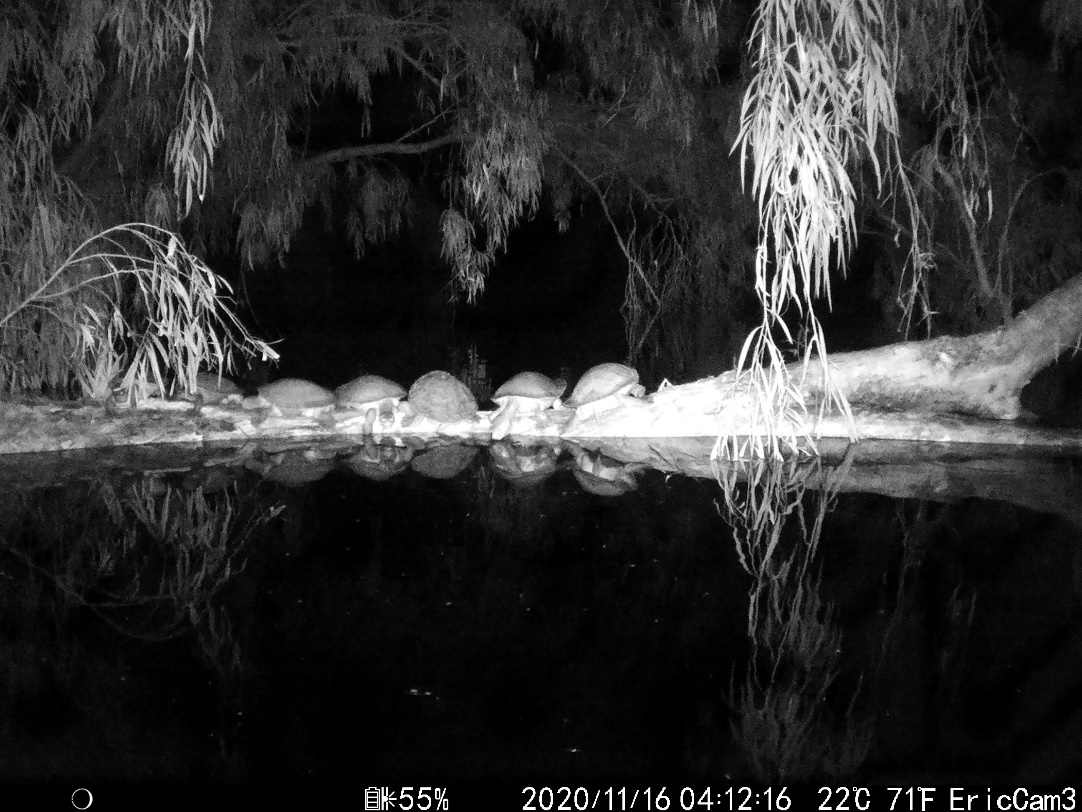


Supplemental Figure 1. Example photos from each of our three cameras used to monitor basking behavior in the wild. Site 1 (top), site 2 (middle) and site 3 (bottom).


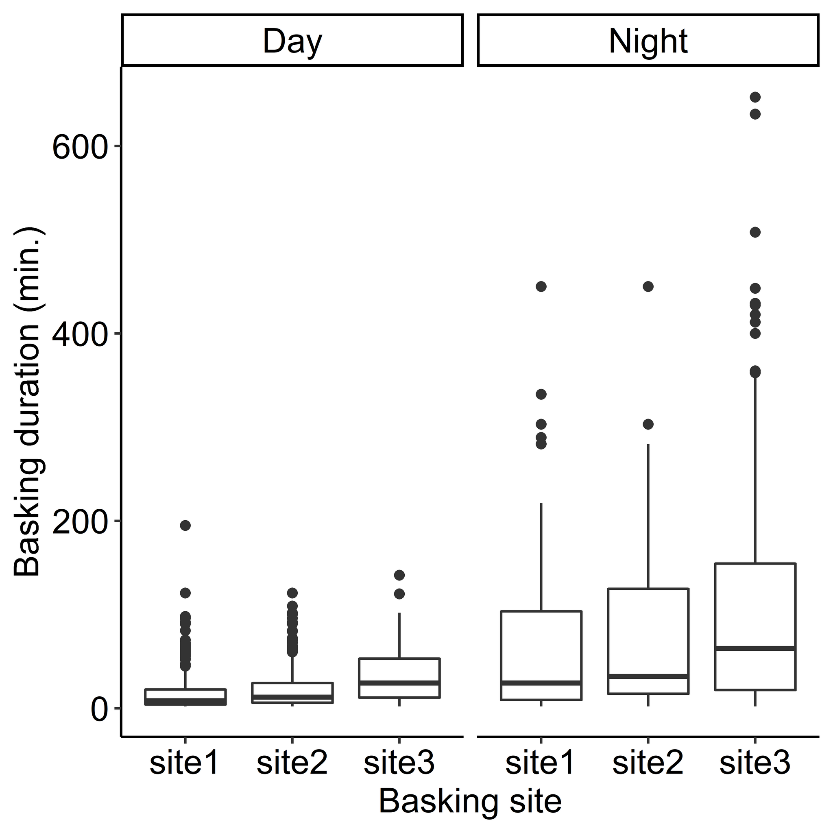


Supplemental Figure 2. Basking durations from each of three trail cameras we deployed in the Ross River, Townsville (each camera was at a different basking site). Basking durations are separated into “Day” and “Night” based on sunrise and sunset times. An additional 55 basking events that transitioned between day and night were not included (see text). Basking durations were largely similar across cameras.





Supplemental Figure 3. To examine the possibility that our results were artifacts of small sample sizes, we examined the probabilities of getting our results (or results with even fewer leeches removed) under a range of hypothetical success rates, where success was defined as the removal of at least one leech from a turtle. For example, for the long-duration nocturnal experiment (n=13), we asked, “if the true population success rate is 50% [thus each turtle has a 50% chance of losing at least one leech], what is the probability that only 2 out of 13 turtles (our observed results) or fewer would lose at least one leech?” (thus, these are one-tailed probabilities). We used this approach to compare a range of hypothetical success rates to the results of our long-duration nocturnal basking trials (2 out of 13 turtles lost at least one leech), short-duration (20 min) outdoor trials (5 out of 10 turtles lost at least one leech), and short-duration (30 min) indoor basking trials (0 out of 6 turtles lost at least 1 leech). The calculated probabilities of getting our observed number of lost leeches (or fewer) at different hypothetical success rates are shown in blue, and the actual success rates we observed are illustrated with green vertical lines. These results suggest that our conclusions are correct, even though our sample sizes were small. For the long-duration experiment, for example, there would only have been a 1.1% chance of getting our observed results (or a loss of fewer leeches) if the true success rate was a mere 50%. By 60%, that probability dropped to 0.1%, and even 60% success is not particularly high. Thus, even if our observed success rates are incorrect, it is unlikely that the true success rates are high enough to make basking an effective means of removing leeches (at least at the time intervals turtles usually basked at in our system).
